# Adeno‐associated viral vectors encoding anti‐P2X7 nanobodies reduce graft‐versus‐host disease in a humanised mouse model

**DOI:** 10.1002/cti2.70061

**Published:** 2025-11-06

**Authors:** Amal Elhage, Janna H Hadaya, Chloe Sligar, Debbie Watson, Sahil Adriouch, Ronald Sluyter

**Affiliations:** ^1^ Molecular Horizons and School of Science University of Wollongong Wollongong NSW Australia; ^2^ Univ Rouen Normandie, INSERM, U1234, Pathophysiology Autoimmunity and Immunotherapy (PANTHER), Normandie Univ Rouen France

**Keywords:** AAVnano, adeno‐associated virus serotype 8, graft‐versus‐host disease, humanised mice, nanobodies, purinergic receptor

## Abstract

**Objectives:**

Graft‐versus‐host disease (GVHD) is an inflammatory disorder that arises following allogeneic haematopoietic stem cell transplantation. P2X7 is an extracellular ATP‐gated cation channel present on immune cells. P2X7 blockade with small molecule inhibitors impairs GVHD development in a humanised mouse model. This study investigated whether adeno‐associated viral (AAV) vectors encoding nanobodies (Nbs) that block mouse P2X7 (mP2X7) or both mP2X7 and human P2X7 (m/hP2X7) impair GVHD development in this model.

**Methods:**

On Day −21, NOD.Cg‐*Prkdc* ^*scid*^ *Il2rg* ^*tm1Wjl*^/SzJ (NSG) mice were injected intramuscularly with 10 × 10^10^ viral genomes encoding either green fluorescent protein (GFP), an anti‐mP2X7 Nb or an anti‐m/hP2X7 Nb, or with saline. On Day 0, mice were euthanised or injected intraperitoneally with 10 × 10^6^ human peripheral blood mononuclear cells and monitored thrice weekly for signs of GVHD until the experiment or disease endpoint.

**Results:**

The anti‐m/hP2X7 and anti‐mP2X7 Nbs reduced clinical GVHD and time to disease onset, as well as liver and lung GVHD. Both Nbs reduced liver human T helper (Th)17 cells. Sera collected at Day 0 and disease endpoint from treated mice, but not from control mice, completely blocked P2X7 activity in human RPMI 8226 and/or murine J774 cells, confirming circulating anti‐P2X7 Nbs in mice from Day 0 to disease endpoint.

**Conclusion:**

This study indicates that P2X7 blockade with an anti‐m/hP2X7 and to a lesser extent an anti‐mP2X7 Nb reduces GVHD progression in humanised mice. This supports the future testing of these P2X7 biologics as a prophylactic therapy for GVHD.

## Graphical abstract

In this study, we found that an adeno‐associated viral vector delivering anti‐P2X7 nanobodies delayed disease onset and reduced clinical, liver and lung disease in a humanised mouse model of graft‐versus‐host disease. These changes were associated with a reduction in human T helper 17 cells. This study highlights the potential of anti‐P2X7 nanobodies as a GVHD prophylaxis.

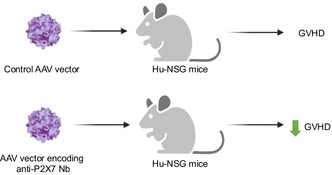

## Introduction

Allogeneic haematopoietic stem cell transplantation (alloHSCT) is a standard treatment for blood cancers, such as leukaemias and lymphomas.^1^ However, a significant complication of alloHSCT is graft‐versus‐host disease (GVHD), a severe inflammatory condition that can lead to tissue damage in the liver, lung, skin and gut in many recipients.^2^ GVHD is driven by donor T cells, which initiate an extensive inflammatory reaction against host organs. It occurs when host (and donor) antigen‐presenting cells (APCs) stimulate donor T cells, leading to their activation and proliferation.^3^ Presently, GVHD prophylaxis involves the administration of different combinations of immunosuppressive agents, including post‐transplant cyclophosphamide.^4^ However, because of the high mortality rate associated with GVHD, novel therapies are still required.

Activation of the ligand‐gated cation channel P2X7 occurs in response to extracellular adenosine 5′‐triphosphate (ATP), triggering a range of pro‐inflammatory responses in various cell types.^5^ These responses are implicated in inflammatory conditions, including GVHD.^6^ The involvement of P2X7 in GVHD pathophysiology and progression has been shown in a humanised mouse model using small molecule antagonists^7^, ^8^, ^9^ and a species‐specific anti‐human (h) P2X7 monoclonal antibody (mAb).^10^ Use of these agents in this model reduces clinical and histological disease. However, improvements in survival have not been observed, and none of these agents in their current form are suitable for clinical translation. The effects of other biologics targeting P2X7 in humanised mice or other GVHD models remain unexplored.

Nanobodies (Nbs) targeting P2X7 have emerged as promising therapeutic agents for a variety of inflammatory and immune‐mediated disorders.^11^ These small, single‐domain antibodies derived from camelid heavy‐chain antibodies possess several advantages, including high specificity and affinity for their target, as well as enhanced tissue penetration.^12^, ^13^, ^14^ When delivered using a single intramuscular (i.m.) injection of an adeno‐associated viral (AAV) vector, Nbs can achieve sustained expression in mice,^15^ offering a potential long‐term therapeutic solution. AAV vectors encoding anti‐mouse P2X7 (mP2X7) Nb (13A7)^12^ or anti‐mouse/human P2X7 (m/hP2X7) Nb (1C81)^16^ are available. These anti‐P2X7 Nbs were found to block P2X7 activity on mouse T cells as early as Day 17 post‐injection.^17^, ^18^ The AAV serotype 8 (AAV8) vector used for P2X7 targeted Nbs in this study, displays a high transduction efficiency and tissue selectivity for muscle and the liver.^19^ However, these biologics are yet to be tested in any humanised mouse model. Using a humanised mouse model of GVHD,^20^, ^21^ this study demonstrates that an AAV8 vector encoding anti‐P2X7 Nbs can reduce clinical GVHD and time to disease onset, as well as histological GVHD. Furthermore, these changes were associated with a reduction in human T helper (Th)17 cells in the livers of mice.

## Results

### The anti‐P2X7 Nbs reduced clinical GVHD and delayed time to disease onset

**Figure 1:**
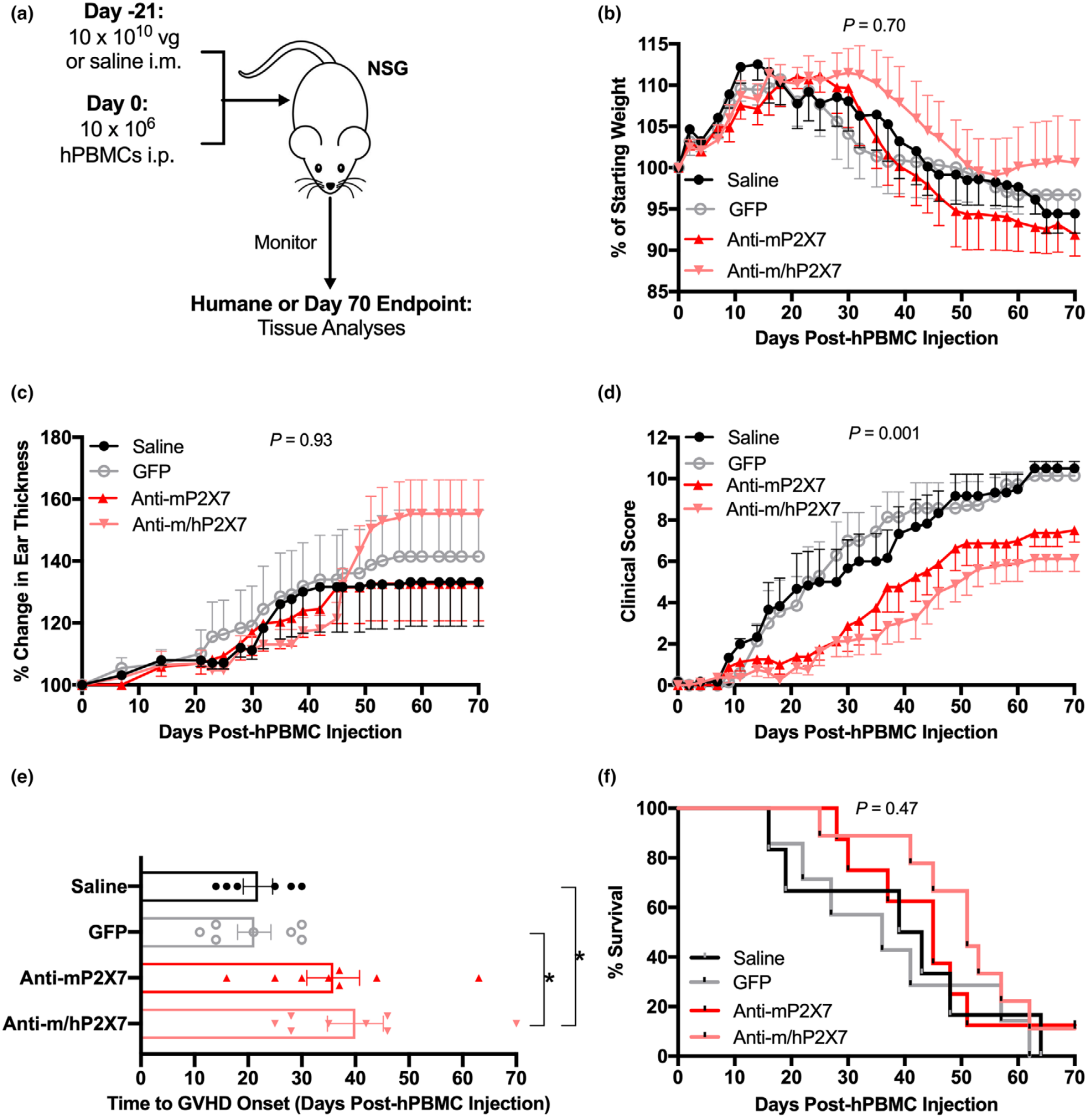
Anti‐P2X7 Nbs reduced clinical graft‐versus‐host disease (GVHD) and delayed time to disease onset. **(a)** Illustration of the humanised mouse model of GVHD. NSG mice (*n* = 6–8 mice for each group) were injected i.m. with adeno‐associated viral (AAV) vector encoding green fluorescent protein (GFP), anti‐mP2X7 Nb or anti‐m/hP2X7 Nb (10 × 10^10^ viral genomes (vg) per mouse) or an equal volume of saline at Day −21 followed by 10 × 10^6^ hPBMCs (*n* = 4 donors) at Day 0. Mice were monitored for up to 70 days for **(b)** weight, **(c)** ear thickness, **(d)** clinical score, **(e)** time to GVHD onset (clinical score of ≥ 3) and **(f)** survival. **(b–e)** Data are represented as the mean ± SEM. **(e)** Symbols represent individual mice. Data pooled from two independent experiments are shown. Significance was assessed by the **(b–d)** two‐way ANOVA with a Tukey's post‐test, **(e)** one‐way ANOVA with a Tukey's post‐test, or **(f)** Mantel–Cox log‐rank test for survival. *P < 0.05.

To investigate whether anti‐P2X7 Nbs can attenuate GVHD in a humanised mouse model, NSG mice were injected i.m. with AAV vectors encoding either green fluorescent protein (GFP), anti‐mP2X7 (13A7) or anti‐m/hP2X7 (1C81) Nbs (10 × 10^10^ viral genomes per mouse), or an equal volume of saline at Day −21. A saline‐treated group was included as a comparison with the AAV vector GFP group, as injection of AAV8 vectors into NSG mice humanised with human peripheral blood mononuclear cells (hPBMCs) had not been reported previously. Mice were then injected with 10 × 10^6^ hPBMCs at Day 0. Mice were monitored thrice weekly from Day −21 and euthanised at humane (disease) or experiment (Day 70) endpoint (Figure 1a). First, weight loss and clinical score were assessed from Day −21 to Day 0, with no significant differences between groups observed (Supplementary figure 1). Post‐hPBMC injection weight loss was observed in saline‐treated and GFP‐treated mice from Day 16 and 21, respectively, (Figure 1b). In contrast, weight loss was observed in anti‐mP2X7 and anti‐m/hP2X7 Nb‐treated mice from Day 28 and 35, respectively. However, there was no significant difference in weight loss between treatment groups (*P* = 0.70).

An increase in ear thickness, indicative of skin GVHD,^9^ was observed in GFP‐treated mice from Day 21 (Figure 1c). In contrast, increases in ear thickness were observed in saline, anti‐mP2X7 and anti‐m/hP2X7 Nb‐treated mice from Day 28 with ear thickness greatest in anti‐m/hP2X7 Nb‐treated mice at endpoint (Figure 1c). However, there were no significant differences in ear thickness between treatment groups (*P* = 0.93).

In contrast to the above, a significant difference in overall clinical score between groups was observed (*P* = 0.001) (Figure 1d). Clinical scores at Day 70 were significantly reduced in anti‐mP2X7 Nb‐treated mice compared to saline‐treated mice (*P* = 0.004) or GFP‐treated mice (*P* = 0.01) (Figure 1d). Clinical scores at Day 70 were further reduced in anti‐m/hP2X7 Nb‐treated mice compared to saline‐treated mice (*P* = 0.0004) or GFP‐treated mice (*P* = 0.0007) (Figure 1d). No significant differences between the two anti‐P2X7 Nb‐treated groups or between the two control groups were observed at any time point.

Time to GVHD onset, defined as the day a mouse reaches and maintains a clinical score total of three or more,^22^ was significantly delayed in anti‐m/hP2X7‐treated mice, but not anti‐mP2X7 Nb‐treated mice, compared to saline‐treated mice (*P* = 0.04) and GFP‐treated mice (*P* = 0.02) (Figure 1e). Time to disease onset was not significantly different between the two anti‐P2X7 Nb‐treated groups.

Differences in clinical score and time to disease onset did not result in significantly increased survival in either anti‐mP2X7 or anti‐m/hP2X7 Nb‐treated mice compared to saline‐treated or GFP‐treated mice (*P* = 0.47). Respective median survival times (MST) were 45, 51, 41 and 36 days (Figure 1f).

### The anti‐m/hP2X7 Nb and to a lesser extent the anti‐mP2X7 Nb reduced histological GVHD at endpoint

**Figure 2:**
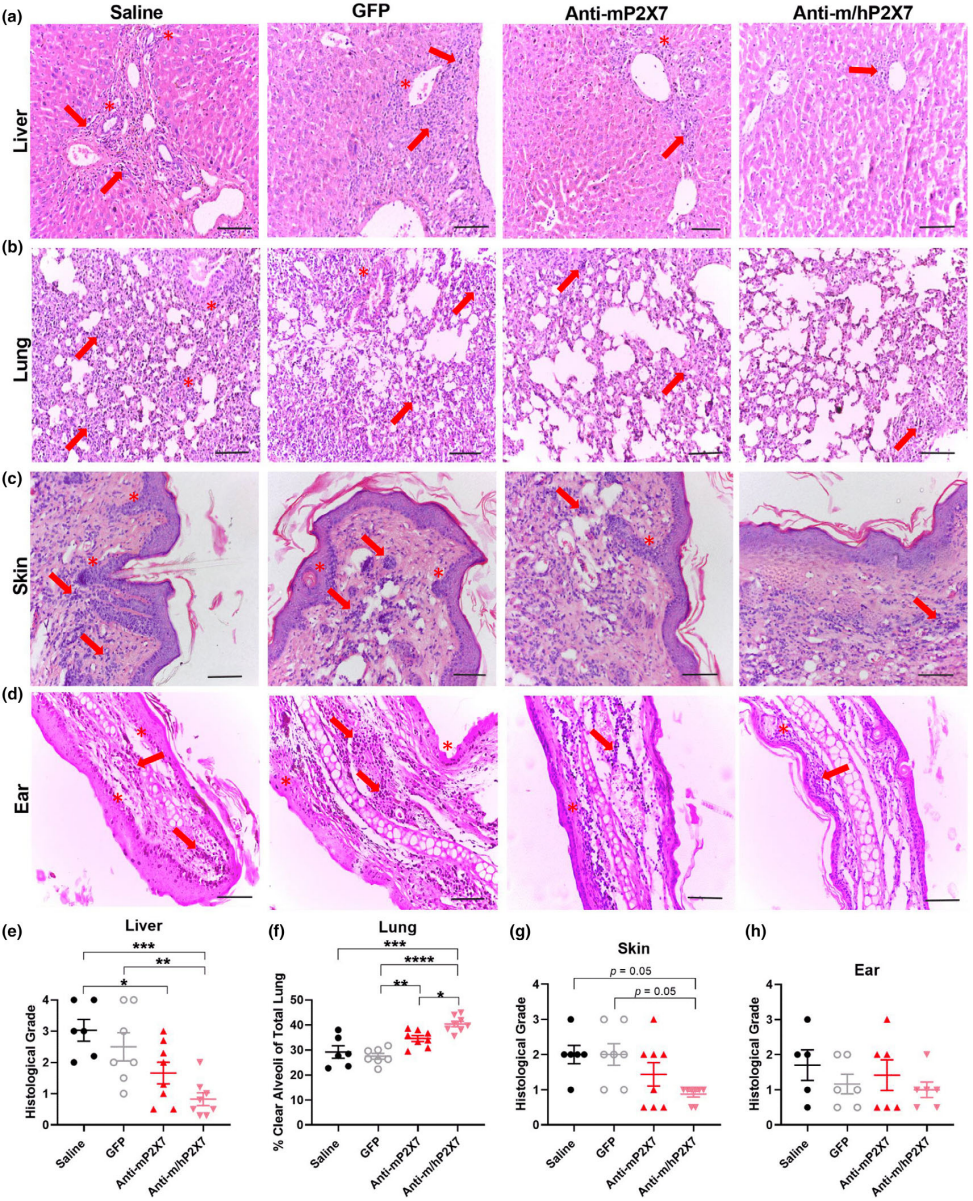
Anti‐m/hP2X7 Nb and to a lesser extent the anti‐mP2X7 Nb reduced histological graft‐versus‐host disease (GVHD) at endpoint. **(a, e)** Livers, **(b, f)** lungs, **(c, g)** skin and **(d, h)** ears from mice treated with saline (*n* = 5–6), green fluorescent protein (GFP) (*n* = 6–7), anti‐mP2X7 Nb (*n* = 6–8) or anti‐m/hP2X7 Nb (*n* = 6–8) were collected at humane (disease) or experiment (Day 70) endpoint. Organs were sectioned (3–5 μm), stained and graded based on evidence of histological GVHD. **(a, e)** Liver, (**c, g**) skin and (**d, h**) ear were measured using a grading system. **(b, f)** Histological GVHD in the lung was measured as the percentage of clear alveoli space of the total lung area. Images representative of at least five mice per treatment group. **(a–d)** Scale bars represent 100 μm. Arrows and asterisks indicate examples of immune cell infiltrates or histological damage, respectively. **(e, h)** Data are represented as the mean ± SEM. Symbols represent individual mice. Data pooled from two independent experiments are shown. Significance was assessed by the **(e, f, h)** one‐way ANOVA with Tukey's post‐test or **(g)** Kruskal–Wallis test with Dunn's multiple comparison correction. *P < 0.05; **P < 0.01; ***P < 0.001; ****P < 0.0001.

Histological GVHD was assessed in the target organs of the mice at either humane (disease) or experiment (Day 70) endpoint (Figure 2) using a histological grading system as described.^23^ Livers from anti‐mP2X7 Nb‐treated mice showed a reduced histological grade compared to saline‐treated mice (*P* = 0.04) but not GFP‐treated mice (Figure 2a and e). Notably, livers from anti‐m/hP2X7 Nb‐treated mice showed a significantly reduced histological grade compared to saline‐treated mice (*P* = 0.0008) and GFP‐treated mice (*P* = 0.009) but not anti‐mP2X7 Nb‐treated mice (Figure 2a and e).

Lung pathology was reduced, with the percentage of clear alveoli space significantly greater in anti‐mP2X7 Nb‐treated mice than in GFP‐treated mice (*P* = 0.01) but not saline‐treated mice (Figure 2b and f). Notably, the percentage of clear alveoli space was significantly greater in anti‐m/hP2X7 Nb‐treated mice than in saline‐treated mice (*P* = 0.0001), GFP‐treated mice (*P* < 0.0001) and anti‐mP2X7 Nb‐treated mice (*P* = 0.03) (Figure 2b and f).

The histological grade in the skin was reduced in anti‐m/hP2X7 Nb‐treated mice compared to saline‐treated mice (*P* = 0.05) and GFP‐treated mice (*P* = 0.05) but not anti‐mP2X7 Nb‐treated mice (Figure 2c and g). In contrast, the histological grades of the ears were similar between all four groups (Figure 2d and h).

### The anti‐P2X7 Nbs altered human lymphocyte proportions in the spleen and liver of humanised mice at endpoint

**Figure 3:**
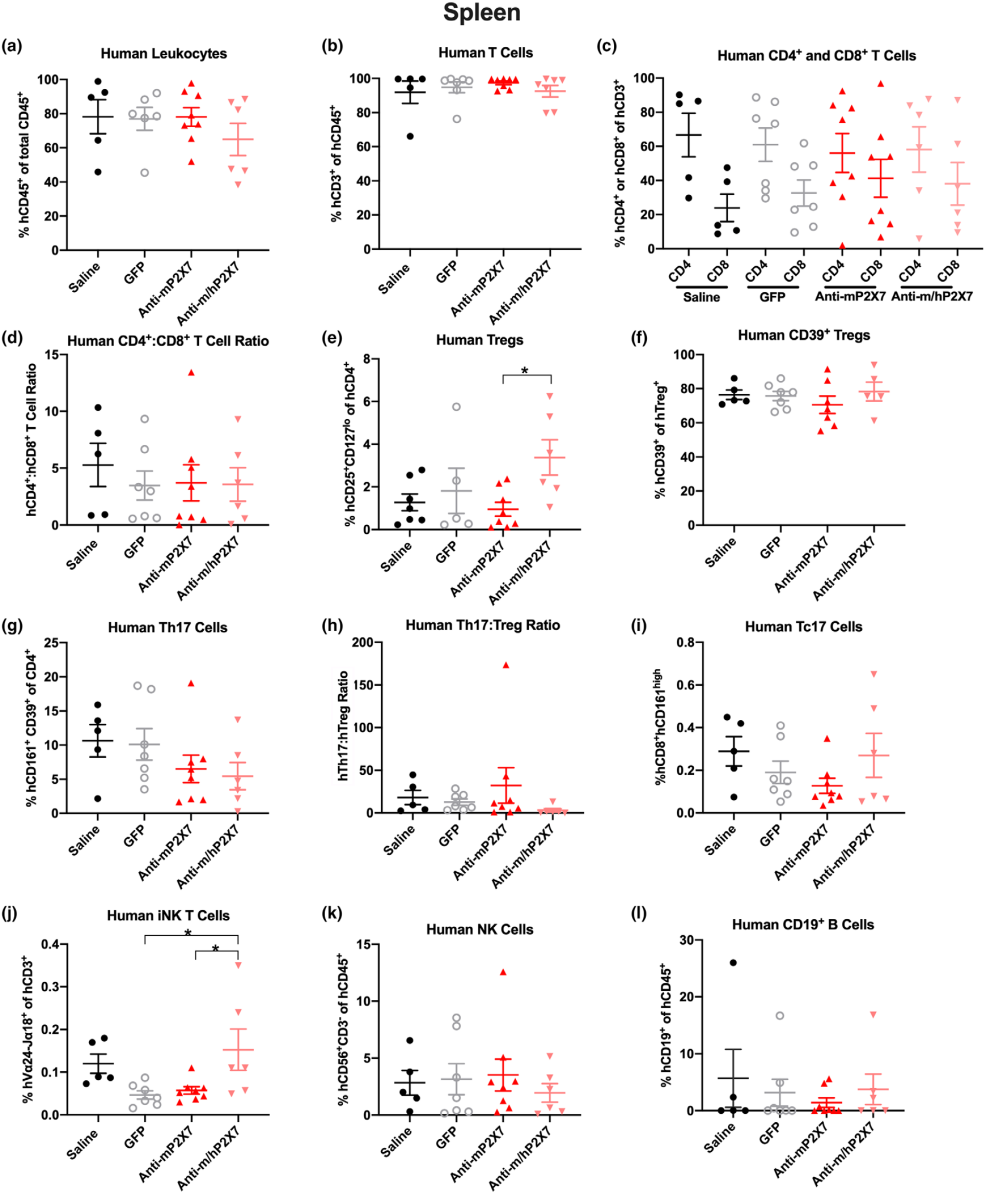
Anti‐m/hP2X7 Nb altered hTreg and hiNK T‐cell proportions in the spleens of humanised mice at endpoint. **(a–l)** Spleens from mice treated with saline (*n* = 5), green fluorescent protein (GFP) (*n* = 7), anti‐mP2X7 Nb (*n* = 8) or anti‐m/hP2X7 Nb (*n* = 6) were collected at humane (disease) or experiment (Day 70) endpoint and immune cell subsets were analysed by flow cytometry. Proportions of **(a)** hCD45^+^ leukocytes were first identified before determining proportions of **(b)** hCD3^+^ T cells, **(c)** hCD4^+^ and hCD8^+^ T cells, **(e)** hCD4^+^hCD25^+^hCD127^lo^ Tregs, **(f)** hCD39^+^ Tregs, **(g)** hCD4^+^hCD161^+^hCD39^+^ Th17 cells, **(i)** hCD8^+^hCD161^high^ hTc17 cells, **(j)** hCD3^+^ hVα24^−^Jα18^+^ hiNK T cells, **(k)** hCD3^−^hCD56^+^ NK cells and **(l)** hCD19^+^ B cells. **(d)** hCD4^+^:hCD8^+^ T‐cell ratios were calculated from **(c). (h)** hTh17:hTreg ratios were calculated from **(e, g). (a–l)** Data are represented as mean ± SEM. Symbols represent individual mice. Data pooled from two independent experiments are shown. Significance was assessed by the **(a, c, e, f, j)** one‐way ANOVA with Tukey's post‐test or **(b, d, g, h, i, k, l)** Kruskal–Wallis test with Dunn's multiple comparison correction. *P < 0.05.

**Figure 4:**
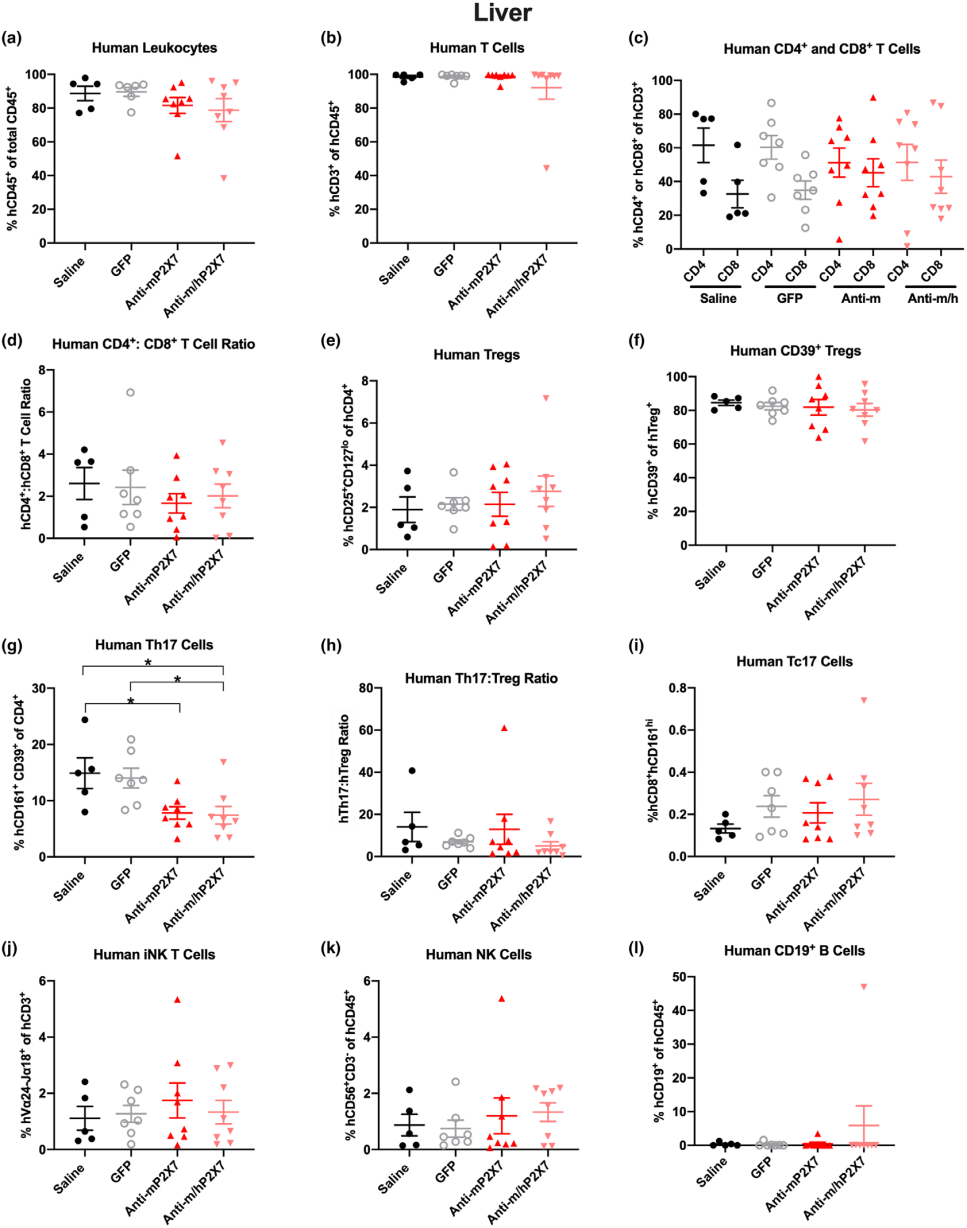
Anti‐P2X7 Nbs altered hTh17 cell proportions in the livers of humanised mice at endpoint. **(a–l)** Livers from mice treated with saline (*n* = 5), green fluorescent protein (GFP) (*n* = 7), anti‐mP2X7 Nb (*n* = 8) or anti‐m/hP2X7 Nb (*n* = 8) were collected at humane (disease) or experiment (Day 70) endpoint and immune cell subsets were analysed by flow cytometry. Proportions of **(a)** hCD45^+^ leukocytes were first identified before determining proportions of **(b)** hCD3^+^ T cells, **(c)** hCD4^+^ and hCD8^+^ T cells, **(e)** hCD4^+^hCD25^+^hCD127^lo^ Tregs, **(f)** hCD39^+^ Tregs, **(g)** hCD4^+^hCD161^+^hCD39^+^ Th17 cells, **(i)** hCD8^+^hCD161^high^ Tc17 cells, **(j)** hCD3^+^hVα24^−^Jα18^+^ iNK T cells, **(k)** hCD3^−^hCD56^+^ NK cells and **(l)** hCD19^+^ B cells. **(d)** hCD4^+^:hCD8^+^ T‐cell ratios were calculated from **(c)**. **(h)** hTh17:hTreg ratios were calculated from **(e, g)**. **(a‐l)** Data are represented as mean ± SEM. Symbols represent individual mice. Data pooled from two independent experiments are shown. Significance was assessed by the **(a‐d, h, i, k, l)** Kruskal–Wallis **(e, f, g, j)** or one‐way ANOVA test with Tukey's post‐test. *P < 0.05.

Spleen and liver human leukocyte proportions in the mice were analysed at either humane (disease) or experiment (Day 70) endpoint by flow cytometry using the consistent gating strategy shown in Supplementary figure 2. Furthermore, given that the ratio of hCD4^+^:hCD8^+^ T cells or hTh17:human T regulatory cells (hTregs) can correlate with GVHD severity in humanised NSG mice,^10^, ^24^ these cell ratios were also examined. In the spleen and liver, no significant differences were observed in the proportions or ratios of any cell types examined between saline‐treated mice and GFP‐treated mice (Figures 3 and 4). Likewise, in the spleen and liver, neither anti‐P2X7 Nb treatment significantly altered proportions or ratios in any of the cell types examined, besides the following. In the spleen, the proportion of hTregs was significantly greater in the anti‐m/hP2X7 Nb‐treated mice than in the anti‐mP2X7 Nb‐treated mice (*P* = 0.04) but not either control group despite the latter three groups having similar proportions of hTregs (Figure 3e). Furthermore, there were no differences in proportions of the highly suppressive hCD39^+^ hTreg subset between the four groups (*P* = 0.60) (Figure 3f). In the spleen, the proportion of invariant (i) natural killer (NK) T cells was significantly increased in anti‐m/hP2X7 Nb‐treated mice compared to GFP‐treated mice (*P* = 0.03) and anti‐mP2X7 Nb‐treated mice (*P* = 0.04), but not saline‐treated mice (*P* = 0.82) (Figure 3k). In the liver, the proportion of hTh17 cells was significantly reduced in anti‐mP2X7 Nb‐treated mice (*P* = 0.04) (Figure 4g) and anti‐m/hP2X7 Nb‐treated mice (*P* = 0.03) compared to saline‐treated mice. Furthermore, the proportion of hTh17 cells in anti‐m/hP2X7 Nb‐treated mice was significantly reduced compared to GFP‐treated mice (*P* = 0.04) (Figure 4g). Despite significant changes in splenic hTregs and liver hTh17 cells, no significant differences were observed between the hTh17:hTreg ratio in either spleens or livers (Figures 3h and 4h, respectively).

### The anti‐P2X7 Nbs did not alter mouse leukocyte proportions in the spleen and liver of humanised mice at endpoint

Despite the humanised mouse model being extensively used by our groups,^25^ we have not examined mouse leukocytes (other than total mCD45^+^ leukocytes) in this model previously. Therefore, spleen and liver mouse leukocyte proportions in the mice were analysed at either humane (disease) or experiment (Day 70) endpoint by flow cytometry using the consistent gating strategy shown in Supplementary figure 3 and as based on prior studies.^26^, ^27^, ^28^ As NSG mice largely lack lymphocytes,^29^ only dendritic cells (DCs), macrophages and myeloid‐derived suppressor cells (MDSCs) were examined, with MDSCs separated as monocytic (M‐) and polymorphonuclear (PMN‐) MDSCs. Furthermore, given P2X7 activation can upregulate the co‐stimulatory molecules CD80 and/or CD86 on myeloid cells *in vitro* ^30^ and in an allogeneic mouse model of GVHD,^31^ the relative amounts of these cell surface molecules on each cell type were examined (Supplementary figure 4).

In the spleen and liver, DCs represented < 1% of the mouse leukocytes present in the spleen and liver (Supplementary figure 5a and 6a). Macrophages and M‐MDSCs each comprised approximately 20% of mouse leukocytes present in these tissues (Supplementary figures 5d, g and 6d, g). PMN‐MDSCs represented the majority of the remaining mouse leukocytes in the spleen and liver (Supplementary figures 5j and 6j). There were no significant differences in the proportions of any of these mouse leukocyte subtypes between any of the four treatment groups (Supplementary figures 5 and 6). Moreover, there were no significant differences between the relative expression of CD80 or CD86 on DCs, macrophages or MDSCs from these tissues between any of the four treatment groups (Supplementary figures 5 and 6), besides one exception, with the relative expression of CD86 significantly greater on splenic M‐MDSCs in anti‐h/mP2X7 Nb‐treated mice compared to splenic M‐MDSCs in anti‐mP2X7 Nb‐treated mice but not on these cells from either control‐treated mice (Supplementary figure 5i).

### The anti‐P2X7 Nbs do not affect human cytokine concentrations in sera of humanised mice at endpoint

Cytokines are crucial in driving the progression of GVHD.^32^ To assess whether amounts of human cytokines in mouse sera varied between treatment groups, a flow cytometric human Th1 LEGENDplex Kit was employed to measure 12 cytokines. Overall, there were no significant differences in concentrations for each of the different cytokines between the four groups (Supplementary figure 7). Near similar concentrations of human interleukin (IL)‐5, IL‐9, IL‐10, IL‐13 and interferon (IFN)γ were observed between groups (Supplementary figure 7c, e–g and k). In contrast, concentrations of IL‐2, IL‐4, IL‐6, IL‐17A, IL‐17F, IL‐22 and tumor necrosis factor‐alpha (TNFα) (Supplementary figure 7a, b, d, h–j and l) were more variable between some groups.

### Circulating anti‐P2X7 Nbs were present in mice injected with AAV vectors encoding anti‐P2X7 Nbs

**Figure 5:**
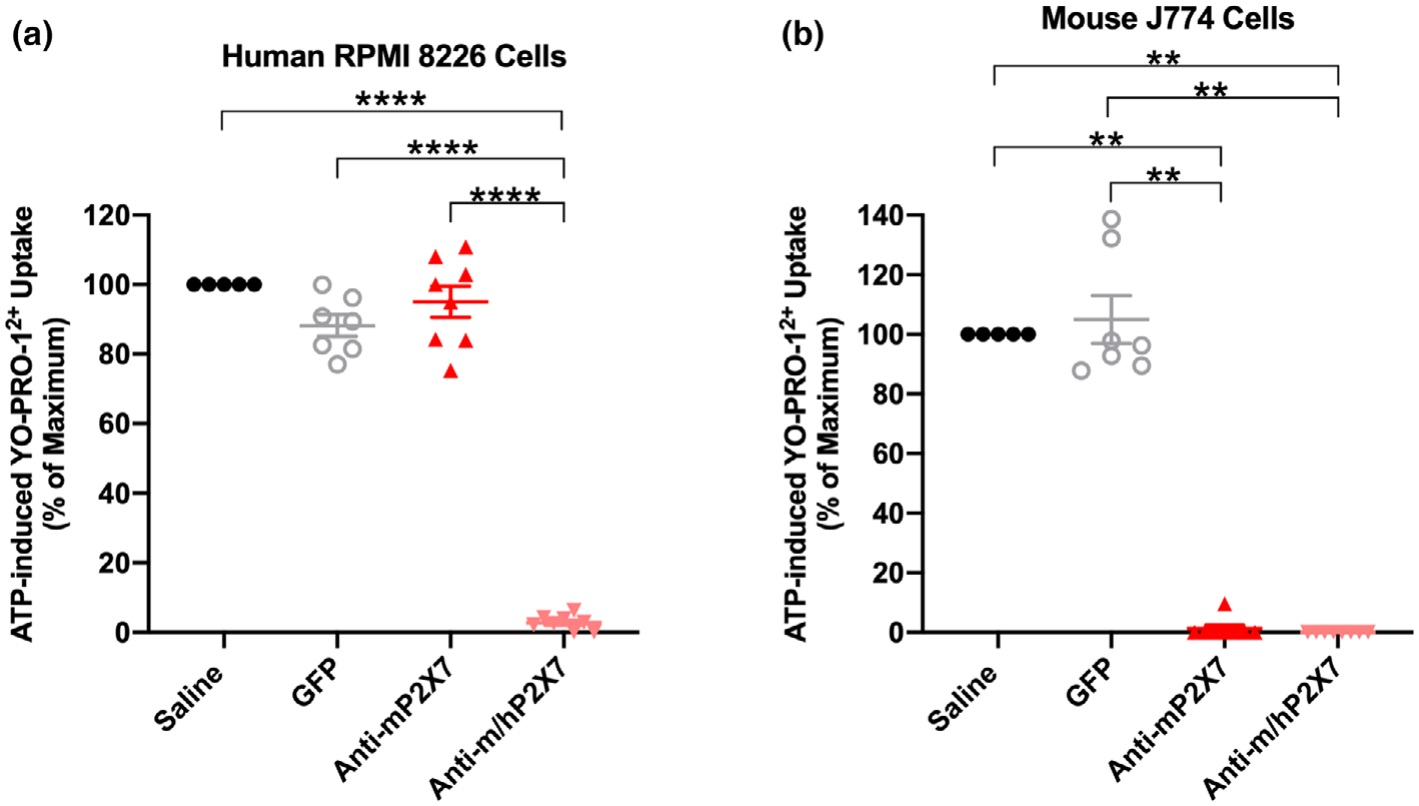
Anti‐mP2X7 and anti‐m/hP2X7 Nbs are present in the sera from humanised mice at endpoint. **(a, b)** Sera from mice treated with saline (*n* = 5), green fluorescent protein (GFP) (*n* = 7), anti‐mP2X7 Nb (*n* = 8) or anti‐m/hP2X7 Nb (*n* = 8) were collected at humane (disease) or experiment (Day 70) endpoint. **(a)** Human RPMI 8226 and **(b)** mouse J774 cells in 90 μL low‐divalent medium (LDM) were pre‐incubated with 10 μL of serum at 37°C for 10 min. Then 900 μL of LDM containing 1 μm of YO‐PRO‐1^2+^ was added and cells were incubated in the absence or presence of 1 mM ATP at 37°C for a further 5 min. Incubations were terminated by the addition of ice‐cold medium containing MgCl_2_ and centrifugation. YO‐PRO‐1^2+^ uptake was then analysed by flow cytometry. ATP‐induced YO‐PRO‐1^2+^ uptake was calculated as the difference in YO‐PRO‐1^2+^ in the presence and absence of ATP and normalised to the ATP response in the presence of sera from saline‐treated mice. **(a, b)** Data are represented as mean ± SEM. Symbols represent individual mice. Data pooled from three independent experiments are shown. Significance was assessed by a one‐way ANOVA with Tukey's post‐test. **P < 0.01; ****P < 0.0001.

To determine whether there were anti‐P2X7 Nbs present in the sera of mice at humane (disease) or experiment (Day 70) endpoint, human RPMI 8226 cells and mouse J774 cells which possess functional P2X7,^33^, ^34^ were pre‐incubated with sera from mice and P2X7 activity was then assessed by flow cytometric measures of ATP‐induced YO‐PRO‐1^2+^ uptake. Preincubation of human RPMI 8226 cells with sera from mice injected with the AAV vector encoding the anti‐m/hP2X7 Nb almost completely inhibited P2X7 activity compared to sera from mice injected with saline (*P* < 0.0001) or the AAV vector encoding either GFP (*P* < 0.0001) or the anti‐mP2X7 Nb (*P* < 0.0001) (Figure 5a). Preincubation of mouse J774 cells with sera from mice injected with the AAV vector encoding the anti‐m/hP2X7 Nb completely inhibited P2X7 activity compared to sera from mice injected with saline (*P* = 0.001) or the AAV vector encoding GFP (*P* = 0.004) (Figure 5b). Likewise, mouse J774 cells pre‐incubated with sera from mice injected with the AAV vector encoding the anti‐mP2X7 Nb completely inhibited P2X7 activity compared to sera from mice injected with saline (*P* = 0.003) or the AAV vector encoding GFP (*P* = 0.009) (Figure 5b). Together, these data confirm the presence of circulating anti‐P2X7 Nbs in humanised mice at humane (disease) or experiment (Day 70) endpoint.

**Figure 6:**
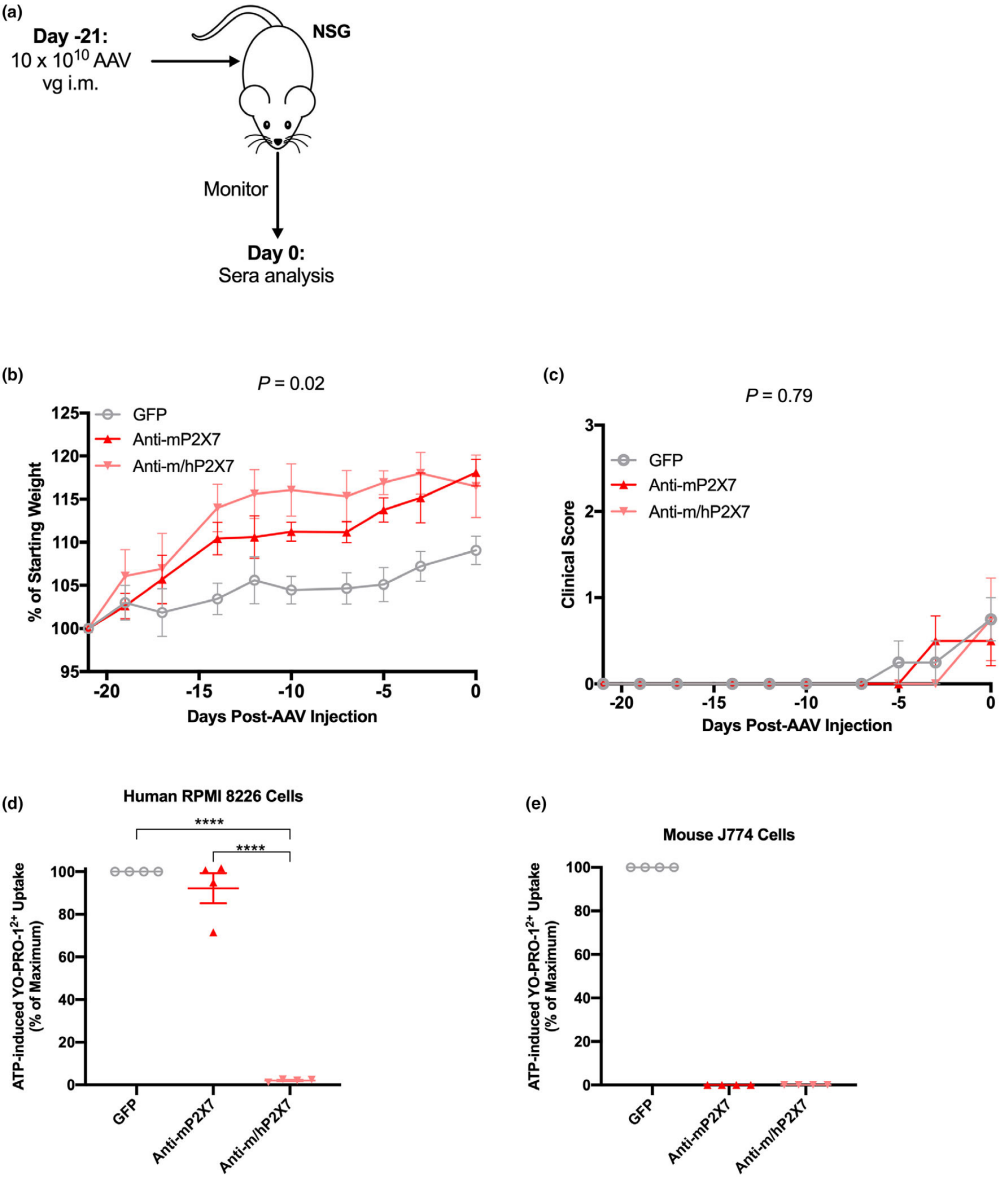
Anti‐mP2X7 and anti‐m/hP2X7 Nbs are present in the sera of non‐humanised mice at Day 0. **(a)** Illustration of non‐humanised NSG mouse model. NSG mice (*n* = 4 mice for each group) were injected i.m. with AAV vector encoding green fluorescent protein (GFP), anti‐mP2X7 Nb or anti‐m/hP2X7 Nb (10 × 10^10^ viral genomes (vg) per mouse) at Day −21. Mice were monitored for 21 days for **(b)** weight and **(c)** clinical score, and sacrificed on Day 0 and sera collected. **(d)** Human RPMI 8226 and **(e)** mouse J774 cells in 90 μL LDM were pre‐incubated with 10 μL of serum at 37°C for 10 min. Then 900 μL of LDM containing 1 μm of YO‐PRO‐1^2+^ was added and cells were incubated in the absence or presence of 1 mM ATP at 37°C for a further 5 min. Incubations were terminated by the addition of ice‐cold medium containing MgCl_2_ and centrifugation. YO‐PRO‐1^2+^ uptake was then analysed by flow cytometry. ATP‐induced YO‐PRO‐1^2+^ uptake was calculated as the difference in YO‐PRO‐1^2+^ in the presence and absence of ATP and normalised to the ATP response in the presence of sera from GFP‐treated mice. **(b–e)** Data are represented as the mean ± SEM. **(d, e)** Symbols represent individual mice. Data are from one independent experiment. Significance was assessed by **(b, c)** two‐way ANOVA with Tukey's post‐test or **(d, e)** one‐way ANOVA test with Tukey's post‐test. ****P < 0.0001.

To determine whether anti‐P2X7 Nbs were present in the sera of mice at Day 0 (day of hPBMC injection), NSG mice were injected i.m. with AAV vectors encoding either GFP, anti‐mP2X7 or anti‐m/hP2X7 Nbs (10 × 10^10^ viral genomes per mouse at Day −21). Mice were monitored thrice weekly for weight loss and clinical score, and euthanised at Day 0 and sera were collected (Figure 6a). To implement the 3Rs,^35^ the saline‐injected group was omitted to reduce mouse numbers. Weight gain was observed in all three groups over the 21‐day period (Figure 6b). In contrast to the first study (Supplementary figure 1), weight was significantly different between groups over this period (*P* = 0.02). Weight was significantly greater between mice injected with AAV vectors encoding either the anti‐mP2X7 Nb (*P* = 0.02) or anti‐m/hP2X7 Nb (*P* = 0.03) compared to those injected with the AAV vector encoding GFP (Figure 6b). This occurred despite co‐housing mice from the different treatment groups in the same cages as undertaken in the first study. In contrast, no difference was observed in clinical score between treatment groups (Figure 6c).

As observed at humane (disease) or experiment (Day 70) endpoint (Figure 5), preincubation of human RPMI 8226 cells with sera collected from mice injected with AAV vectors encoding the anti‐m/hP2X7 Nb 21 days post‐injection almost completely inhibited P2X7 activity compared to sera from mice injected with AAV vectors encoding either GFP (*P* < 0.0001) or the anti‐mP2X7 Nb (*P* < 0.0001) (Figure 6d). Again, preincubation of mouse J774 cells with sera collected from mice injected with the AAV vector encoding the anti‐m/hP2X7 Nb 21 days post‐injection completely inhibited P2X7 activity compared to sera from mice injected with the AAV vector encoding GFP (Figure 6e). Likewise, preincubation of mouse J774 cells with sera collected from mice injected with the AAV vector encoding the anti‐mP2X7 Nb 21 days post‐injection completely inhibited P2X7 activity compared to sera from mice injected with the AAV vector encoding GFP. Because of all groups with J774 cells having a standard error of zero, statistical differences could not be determined (Figure 6e). Together, these data confirm the presence of circulating anti‐P2X7 Nbs in non‐humanised NSG mice injected with AAV vectors encoding either anti‐P2X7 Nb at Day 0 (21 days post‐AAV vector injection).

## Discussion

The aim of this study was to determine whether anti‐P2X7 Nbs, delivered following a single i.m. injection of an AAV vector (Day −21), can reduce disease in a humanised mouse model of GVHD. The anti‐m/hP2X7 Nb, and to a lesser extent the anti‐mP2X7 Nb, reduced clinical GVHD and histological GVHD in the liver and lungs at humane (disease) or experiment (Day 70) endpoints. These effects corresponded to a decrease in liver hTh17 cells in both anti‐P2X7 Nb groups, and to some extent an increase in splenic hTregs and hiNK T cells in the anti‐m/hP2X7 Nb group. Furthermore, the presence of circulating anti‐P2X7 Nbs was confirmed at Day 0 and at humane or experiment (Day 70) endpoints.

This study demonstrates the ability of anti‐P2X7 Nbs to attenuate clinical and histological GVHD in a humanised mouse model of this disease. Furthermore, the results reveal greater efficacy of the anti‐m/hP2X7 Nb (1C81) than the anti‐mP2X7 Nb (13A7) supporting the concept that both host (mouse) and donor (human) P2X7 are involved in GVHD development. Whether this reflects differences in pharmacokinetics or affinity between the two Nbs remains unknown, although these Nbs display similar IC_50_ values (2.0 and 2.8 nM for 1C81 and 13A7, respectively)^17^, ^36^ Of note, the anti‐P2X7 Nb treatment, especially with the anti‐h/mP2X7 Nb, reduced histological GVHD in the liver and lungs, with a similar but non‐significant reduction in the skin. Previously our groups have observed a significant reduction in the livers of humanised mice treated with the P2X7 antagonist Brilliant Blue G (BBG)^7^, ^8^, ^9^ and in the livers and lungs of humanised mice treated with the anti‐hP2X7 mAb.^10^ Thus, the anti‐h/mP2X7 Nb is the most effective P2X7 inhibitor in reducing histological GVHD in humanised mice to date. Differences between the anti‐h/mP2X7 Nb and BBG may reflect increased Nb tissue penetration or sustained production of Nb compared to BBG resulting in greater or extended blockade of P2X7.

Prior to this study, targeting of P2X7 in pre‐clinical models of GVHD was restricted to five different small molecule P2X7 antagonists or an anti‐human P2X7 mAb,^7^, ^8^, ^9^, ^10^, ^31^, ^37^, ^38^, ^39^, ^40^ none of which is suitable for use in a clinical setting for GVHD, with the exception of stavudine. This study offers a new therapeutic approach to targeting P2X7 in GVHD. AAV vectors are increasingly being utilised as a gene therapy because of their efficiency and safety in delivering genes of interest into cells and producing therapeutic proteins *in vivo*, both in animal models as well as in humans.^41^, ^42^ Because of their strong safety profile, AAV vectors have been used in numerous clinical trials, with six AAV vectors approved for use in neurological or inflammatory disorders by the Food and Drug Administration (FDA) or European Medicines Agency (EMA) to date.^19^, ^43^ Given these regulatory approvals and the lack of clinical adoption of P2X7 antagonists to date,^44^ there is potential for testing anti‐P2X7 Nbs, either administered as proteins or delivered via AAV vectors in clinical settings and for future clinical adoption.

The current study demonstrated the presence of circulating anti‐P2X7 Nbs in sera of mice from Day 21 to Day 91 post‐AAV vector injection. These data largely align with previous studies. Nbs are detectable in the sera of mice as early as Day 7 post‐AAV vector injection, with the concentration slowly increasing until reaching a plateau at 3–4 weeks post‐AAV vector injection.^15^ This former study also showed the systemic expression of Nbs to at least Day 120. Another study demonstrated AAV‐delivered P2X7 Nbs reached concentrations of ~30 μg mL^−1^ at Day 17 and increased over time before plateauing at ~100 μg mL^−1^ between Days 30 and 60, which was maintained over time until Day 107.^17^ A possible disadvantage of AAV vector therapies to deliver anti‐P2X7 Nbs is the potentially irreversible or prolonged blockade of P2X7 *in vivo*, without the ability to halt the production of Nbs and therefore treatment. This may be problematic in some instances in a clinical setting. Prolonged P2X7 blockade may lead to unforeseen physiological effects in mice or humans, such as immune dysregulation, compromised barrier functions or alterations in the microbiota.^15^ This limitation could be potentially overcome by using an inducible or reversible on‐switch AAV vector system.^45^

Leukocyte profiling revealed that the reduction in clinical and histological GVHD by the anti‐m/hP2X7 Nb corresponded to a reduction in hTh17 cells in the liver and to some extent an increase in hTregs and iNK T cells in the spleen. These findings mirror those in humans after alloHSCT, where increased proportions of hTregs and iNK T cells^46^, ^47^, ^48^ and decreased proportions of hTh17 cells are associated with decreased GVHD severity.^49^, ^50^ In addition, hTregs and hiNK T cells have been shown to reduce GVHD severity in humanised NSG mice.^51^, ^52^ Conversely, hTh17 cells can exacerbate GVHD in these mice.^53^, ^54^ Therefore, it is probable that the anti‐m/hP2X7 Nb reduced GVHD progression by altering these human lymphocyte subsets. In contrast, the reduction in clinical and histological GVHD by the anti‐mP2X7 Nb corresponded to a reduction in liver hTh17 cells only. P2X7 blockade can influence the differentiation of naïve CD4^+^ T cells, promoting the development of Tregs while inhibiting Th17 cell differentiation to decrease the Th17:Treg ratio.^54^ This provides a potential explanation for the beneficial effects of the anti‐P2X7 Nbs observed in the current study; however, the hTh17:hTreg ratio was not significantly altered by the anti‐P2X7 Nbs in this study. Since P2X7 activation on DCs can promote Th17 cell differentiation^55^, ^56^ it is possible that the anti‐P2X7 Nbs may have inhibited P2X7 activation on DCs to impair Th17 cell differentiation *in vivo*. Given that both the anti‐mP2X7 and anti‐m/hP2X7 Nbs reduced hTh17 cells, this suggests that mouse (host) DCs may be involved in this process as well as human (donor) DCs, with the latter in line with the reduction in hTh17 cells observed in this model following treatment with anti‐hP2X7 mAb.^10^

In further contrast to the observed effects on hTregs and hTh17 cells, the anti‐P2X7 Nbs did not alter the circulating concentrations of human cytokines, including IL‐10 and IL‐17, the two cytokines associated with these respective cell types. Moreover, hIFNγ was not different between any groups, contrary to previous reductions in this cytokine following treatment with BBG in this model.^7^, ^9^ Given that host (mouse) and donor (human) P2X7 were both potentially blocked by BBG (in these former studies) and by the anti‐m/hP2X7 Nb (in the current study), it is possible that BBG reduces hIFNγ concentrations by blocking off‐target molecules, such as pannexin‐1,^57^ to prevent the subsequent release of ATP from cells in humanised mice.

This study contains limitations. First, histological and flow cytometric analyses were conducted at different timepoints, which limits direct comparison across datasets. Standardising analyses at a single predefined endpoint in future studies may improve comparisons between groups. Second, mucosal‐associated invariant T (MAIT) cells, which can also express CD161,^58^ were not excluded from the Tc17 cell population. This raises the possibility that the CD3^+^CD8^+^CD161^+^ cells in this study represent Tc17 and/or MAIT cells. Third, one or two points were absent in some groups for some flow cytometric cell and cytokine analyses because of insufficient sample amounts recovered from those mice. This potentially obscured some minor differences between groups.

## Conclusions

In conclusion, this study demonstrated that the anti‐m/hP2X7 Nbs, and to a lesser extent the anti‐mP2X7 Nbs, reduced clinical and histological GVHD in a humanised mouse model. These changes were largely associated with a reduction in hTh17 cells in the livers of mice. Collectively, this study supports the future testing of these P2X7 biologics as a prophylactic therapy for GVHD in alloHSCT recipients.

## Methods

### Cell lines

Human multiple myeloma RPMI 8226 cells were obtained from the European Collection of Cell Cultures (Salisbury, UK), and mouse macrophage J774 cells were obtained from the American Type Culture Collection (Manassas, USA). Both lines were maintained in RPMI‐1640 medium containing 10% foetal calf serum (FCS) and 2 mM GlutaMAX (Thermo Fisher Scientific, Waltham, USA). J774 cells were harvested by mechanical scraping. Cell lines were assessed for *Mycoplasma* spp. contamination using the Myco Alert Mycoplasma Detection Kit (Lonza, Basel, Switzerland) as per the manufacturer's instructions and were routinely negative.

### hPBMC isolation

Human blood was collected and utilised in accordance with approval by the University of Wollongong Human Ethics Committee (12/290). hPBMCs were isolated as described.^20^ Briefly, whole blood was collected into Vacutainer heparin tubes (BD Biosciences, San Diego, USA) from healthy donors (two males; two females; age range 22–25 years) and diluted in an equivalent volume of sterile Dulbecco's PBS (D‐PBS) (Thermo Fisher Scientific). Samples were underlaid with Ficoll‐Paque PLUS (GE Healthcare; Uppsala, Sweden) and centrifuged (560× *g* for 30 min with brake disengaged). hPBMCs were recovered from the gradient interface and washed twice with D‐PBS and resuspended at a final concentration of 10 × 10^7^ cells mL^−1^ in D‐PBS for injection into mice.

### Production of recombinant AAV vectors coding for anti‐P2X7 Nbs

The transgenes used for the generation of the plasmids coding for the anti‐P2X7 Nbs have been described previously.^12^ Briefly, the plasmid coding for the construct termed 1C81 used in this study was based on a Nb dimer fused to the Alb8 anti‐albumin Nb. For that, the coding sequences corresponding to two 1C81 Nbs were fused using a 35‐GS linker (GGGGS) × 7. This construct was further fused to the anti‐albumin Nb Alb8 via a 9‐GS linker (GGGGSGGGS).^15^, ^17^, ^18^ The plasmid coding for the construct termed 13A7 was constructed by fusing the sequence corresponding to the Nb 13A7 to the hinge and Fc regions of a mutated mouse IgG_1_ antibody carrying the ‘LSF’ mutations (T252L, T254S and T256F),^12^, ^15^, ^17^, ^18^ used previously to confer a higher affinity to the neonatal Fc receptor and thereby an extended half‐life *in vivo*. To produce AAV8 vectors coding for these anti‐P2X7 Nbs, the construct was recloned into a pFB plasmid under the control of a CBA promoter. Production, purification and titration, were all performed by Virovek (Houston, USA) using the baculovirus expression system in Sf9 insect cells as described previously.^15^, ^17^, ^18^

### Humanised mouse model of GVHD

All animal experiments were conducted in accordance with approval by the University of Wollongong Animal Ethics Committee (AEPR2220). A humanised mouse model of GVHD was used as described.^20^ Female NOD.Cg‐*Prkdc* ^*scid*^ *Il2rg* ^*tm1Wjl*^/SzJ (NSG) mice (aged 6–8 weeks) from the Australian BioResources (Moss Vale, Australia) were acclimatised for 1 week prior to experiments. Mice were injected intramuscularly (i.m.) (caudal thigh) with approximately 10 × 10^10^ viral genomes of AAV8 vectors encoding either GFP, an anti‐mP2X7 Nb (13A7) or anti‐m/hP2X7 Nb (1C81), or an equal volume of saline (D‐PBS) 21 days prior to an intraperitoneally (i.p.) injection of 10 × 10^6^ freshly isolated hPBMCs (Day 0). In one Nb study, mice, which were not injected with hPBMCs, were sacrificed at Day 0. Mice were monitored, in a blinded fashion, at least thrice weekly for weight loss, survival and clinical score from the first day of injections until endpoint (as indicated) using an established scoring system (Table 1). Ear thickness was measured using Interapid spring‐loaded callipers (Rolle, Switzerland) once weekly until disease onset (Day 21), then thrice weekly until humane (disease) or experiment endpoint. Mice were euthanised at humane (disease) endpoint or experiment endpoint (Day 0 or 70) by slow‐fill CO_2_ and blood and organs were obtained for analysis.

**Table 1:** Clinical scoring criteria for the assessment of GVHD in humanised mice

Score^a^	0	1	2	3
Chronic weight loss^b^	< 5%	5 to 9.9%	10 to 14.9%	≥ 15%
Acute weight loss^c^	< 5%	5 to 6.9%	7 to 14.9%	≥ 15% or if ≥ 13% for ≥ 48 h
Activity	Cage exploration prior to removal of lid	Some reduction in activity but stands on hindlimbs and actively explores	Slow to move, explores when shelters are removed	Remains stationary and does not explore
Posture	Normal	Hunching noted at rest only	Hunching noted with movement and hunched back when walking	Severe hunching ‐ very hunched when stationery, nose is close to ground when resting
Fur	Normal coat, smooth and glossy	Mild to moderate ruffling	Severe ruffling, < 50% of surface area with hair loss	≥ 50% of surface area with hair loss
Skin integrity	Normal	Scaling of paws/tail	Scaling at additional areas	Areas of denuded skin with loss of surface layers

^a^ Mice were euthanised if they scored 3 in any category or reached a total score ≥ 10.

^b^ Chronic weight loss was determined as percentage loss from the starting weight (Day 0).

^c^ Acute weight loss was determined as percentage loss from the maximum weight over the previous 7 days.

### Histological analyses on humanised mouse tissues

Organs were removed from euthanised mice and processed as described.^23^ Briefly, samples were fixed with neutral buffered (10%) formalin (Sigma Aldrich, St Louis, USA), embedded in paraffin wax, sectioned (3–5 μm) using an RM2255 microtome (Leica Biosystems; Wetzlar, Germany), placed on frosted glass microscope slides (Universal Choice, Turrella, Australia) and stained with haematoxylin and eosin (POCD, Atarmon, Australia). Sections on slides were mounted with dibutylphthalate polystyrene xylene (Sigma Aldrich) and glass cover slips (Westlab, Mitchell Park, Australia). Histological differences were measured using a Leica DMIL inverted light microscope with a 20× objective. GVHD in the liver, skin and ear was assessed, in a blinded fashion, using a standardised grading system (using grades from 0 to 4) as described.^23^ GVHD in the lung was assessed, in a blinded fashion, as the percent clear alveoli space of the total lung area measured using the Fiji software as described.^23^ At least three representative images of each tissue for each mouse were captured, graded and averaged.

### Immunophenotyping by flow cytometry

Spleens and livers were collected from euthanised mice, and dissociated and immunophenotyped as described.^9^ Briefly, homogenates were filtered through a 70 μm nylon filter (BD Bioscience) and centrifuged (300× *g* for 5 min) before being incubated for 5 min with red cell lysis buffer (150 mM NH_4_Cl, 1 mM KHCO_3_ and 0.1 mM Na_2_CO_3_). Cells were washed and resuspended in D‐PBS (1 × 10^6^ cells tube^−1^). Cells were stained with Zombie Near Infrared (NIR) live per dead dye (BioLegend, San Diego, USA) for 15 min in the dark on ice. Cells were subsequently washed with D‐PBS containing 2% FCS (300× *g* for 5 min). Cells were incubated with fluorochrome‐conjugated mAbs (Supplementary tables 1 and 2) for 15 min in the dark on ice. Samples were washed (300× *g* for 5 min) and resuspended in D‐PBS. Data were acquired using a BD Biosciences LSR Fortessa X‐20 flow cytometer. Proportions of human and mouse immune cell subsets and mean fluorescent intensity (MFI) of mCD80 and mCD86 on mouse leukocytes were assessed using the FlowJo software version 10.7.1 (BD Biosciences).

### Serum cytokine analyses

Blood was collected from euthanised mice and serum was obtained as described.^20^ Cytokine concentrations were determined using a human T Helper‐1 LEGENDplex Kit (BioLegend) as per the manufacturer's instructions using a BD Biosciences Accuri C6 flow cytometer. Data were analysed using the LEGENDplex data analysis software (BioLegend).

### ATP‐induced YO‐PRO‐1^2+^ dye uptake assay to test sera from mice injected with AAV vectors

To assess the presence of anti‐P2X7 Nbs in the sera of humanised mice injected with AAV vectors encoding either GFP, anti‐mP2X7 Nb or anti‐m/hP2X7 Nb, or with saline, P2X7 activity in cells was assessed using a variation of a previously described ATP‐induced YO‐PRO‐1^2+^ dye uptake assay.^10^ Human RPMI 8226 cells and mouse J774 cells were washed thrice with low‐divalent medium (LDM) (145 mM NaCl, 2 mM KCl, 0.2 mM CaCl_2_, 13 mM D‐glucose, 10 mM HEPES, pH 7.5) (300× *g* for 5 min) and resuspended in LDM at 1 × 10^6^ cells 90 μL^−1^. These cells were pre‐incubated with 10 μL of serum from an individual mouse for 10 min at 37°C prior to the addition of 900 μL of LDM containing 1 μm YO‐PRO‐1 iodide (Thermo Fisher Scientific) and incubated in the absence or presence of 1 mM of ATP (Sigma Aldrich). Data were acquired with an Accuri C6 flow cytometer. The MFI of YO‐PRO‐1^2+^ uptake was determined using the FlowJo software v10.7.1. Inhibition of ATP‐induced YO‐PRO‐1^2+^ uptake by sera was calculated as a percent of the control sera (as indicated), which was set as 100% YO‐PRO‐1^2+^ uptake.

### Data presentation and statistics

Data are represented as the mean ± standard error of the mean (SEM). Data were tested for normality using a Shapiro–Wilk test. A one‐way analysis of variance (ANOVA) (parametric) or Kruskal–Wallis (non‐parametric) test was applied with Tukey's *post hoc* test and with Dunn's multiple comparison correction. Weight, clinical score and ear thickness differences in the mice were determined with a two‐way ANOVA with Tukey's *post hoc* test Survival differences were determined by a log‐rank (Mantel–Cox) test. All statistical analyses were conducted and graphs assembled using the GraphPad Prism software v8.0.2. (GraphPad Software; La Jolla, CA, USA). For all analyses, differences were considered significant if *P* < 0.05. Missing data points in cell subset analyses were due to insufficient cells being recovered from the organs impairing reliable quantification of cell proportions. Missing data points in cytokine analyses were due to insufficient blood samples being obtained leading to low serum volumes.

## Author contributions

**Amal Elhage:** Investigation; software; formal analysis; data curation; writing – review and editing; writing – original draft; visualization. **Janna H Hadaya:** Investigation; formal analysis; software; data curation; writing – review and editing. **Chloe Sligar:** Investigation; writing – review and editing. **Debbie Watson:** Conceptualization; funding acquisition; writing – review and editing; methodology; supervision; resources. **Sahil Adriouch:** Conceptualization; funding acquisition; methodology; writing – review and editing; resources. **Ronald Sluyter:** Conceptualization; funding acquisition; writing – review and editing; methodology; project administration; supervision; resources.

## Conflict of interest

The authors have no conflicts of interest to disclose.

## Supporting information


Supplementary data 1

## Data Availability

The data that support the findings of this study are available from the corresponding author upon reasonable request.
