# Supplementary material for: Adeno‐associated viral vectors encoding anti‐P2X7 nanobodies reduce graft‐versus‐host disease in a humanised mouse model
Source: Clin Transl Immunology. 2025 Nov 6;14(11):e70061. doi: 10.1002/cti2.70061 (PMC12591662; doi:10.1002/cti2.70061)
Supplement: Supplementary file 1 — Supplementary data 1 [file CTI2-14-e70061-s001.docx]

**
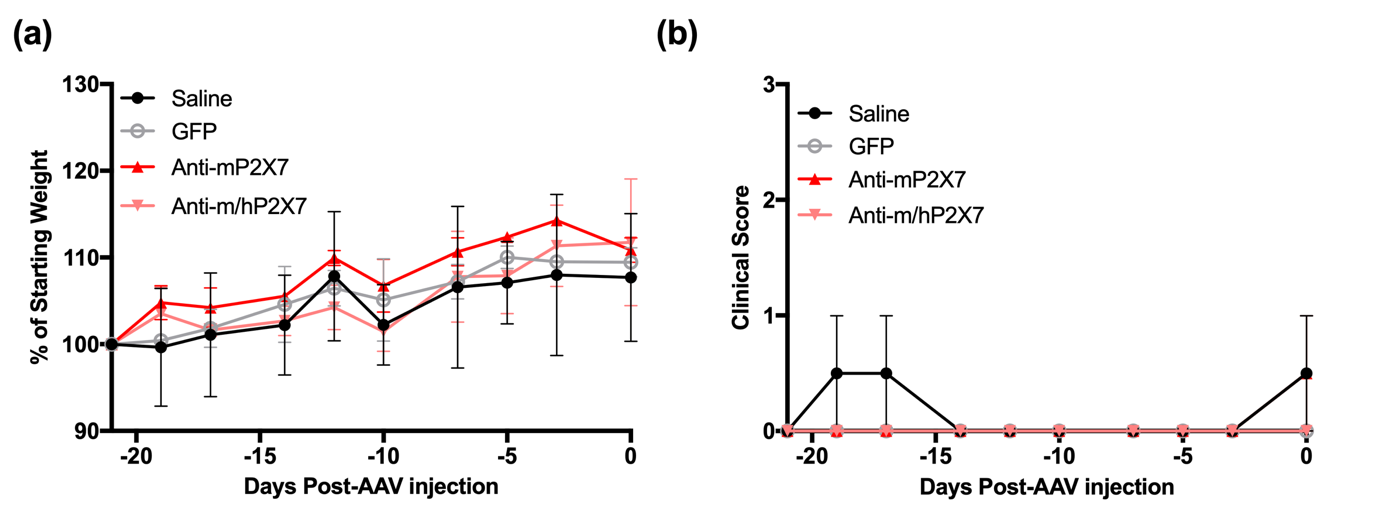
**

**Supplementary Figure 1: Neither the AAV vectors nor anti-P2X7 Nbs impacted weight or clinical score prior to hPBMC injection. (a, b)** NSG mice (*n* = 6-8 mice for each group) were injected i.m. with AAV vector encoding GFP, anti-mP2X7 Nb or anti-m/hP2X7 Nb (10 × 10^10^ viral genomes (vg) per mouse) or an equal volume of saline at Day -21. Mice were monitored thrice weekly until Day 0 for **(a)** weight and **(b)** clinical score. **(a, b)** Data are represented as the mean ± SEM. Data are from two independent experiments. Significance was assessed by two-way ANOVA.

**
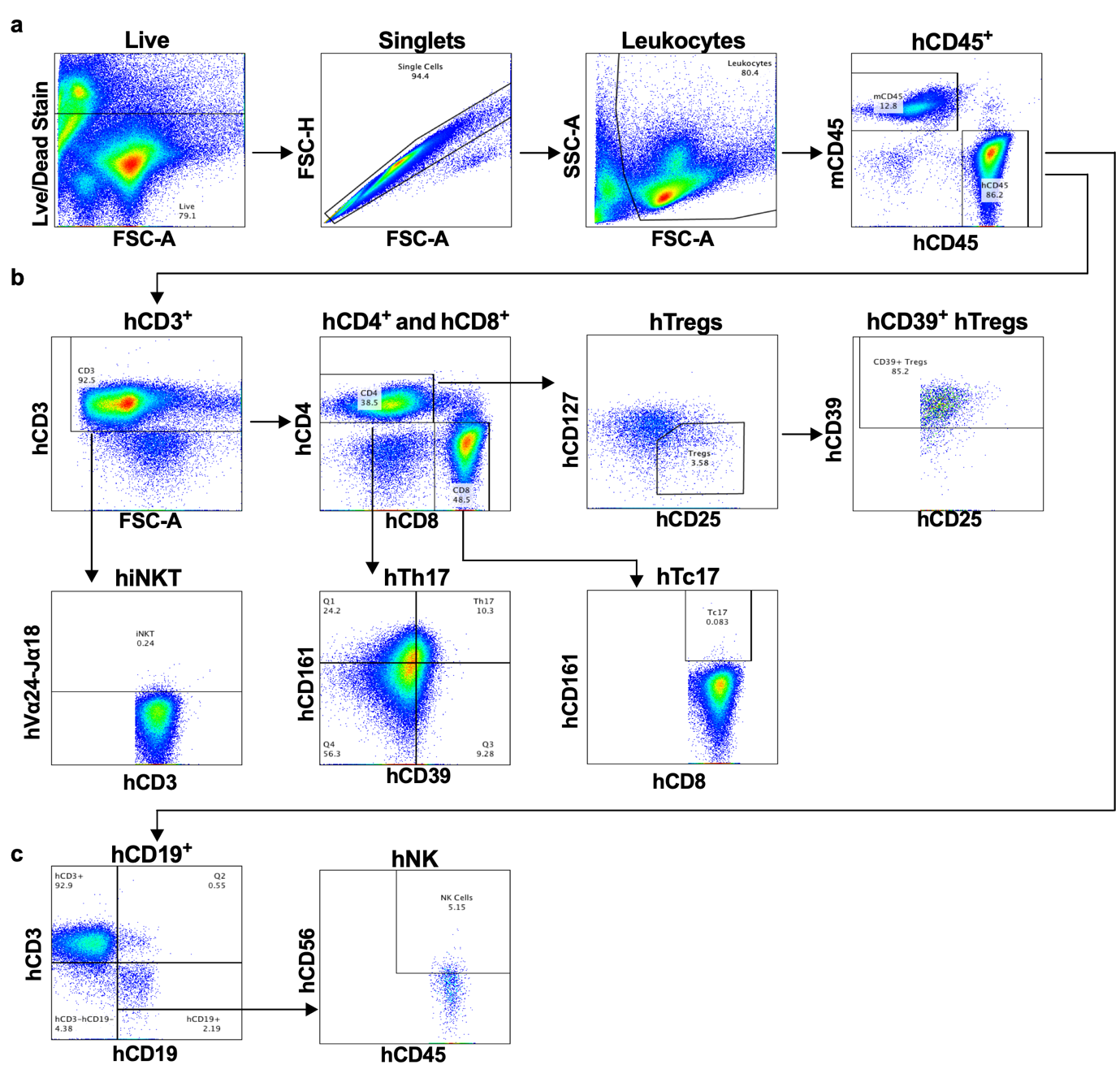
Supplementary Figure 2: Gating strategy used to identify human leukocyte populations*.* (a-c)** Human leukocytes were immunolabelled with mAbs and analysed by flow cytometry. **(a)** Live cells were gated based on forward scatter-area (FSC-A) and Zombie NIR staining. Singlets were gated based on FSC-A and forward scatter-height (FSC-H). Human leukocytes were gated using FSC-A and side scatter-area (SSC-A). The proportion of hCD45^+^ leukocytes was then identified before gating **(b)** hCD3^+^ T cells, hCD4^+^ and hCD8^+^ T cell subsets, hCD4^+^hCD25^+^hCD127^lo^ Tregs, hCD39^+^ Tregs, hCD4^+^hCD161^+^hCD39^+^ Th17 cells, hCD8^+^hCD161^hi^ Tc17 cells, hCD3^+^hVα24^-^Jα18^+^ iNK T cells, or **(c)** hCD19^+^ B cells and hCD3^-^hCD19^-^hCD56^+^ NK cells.


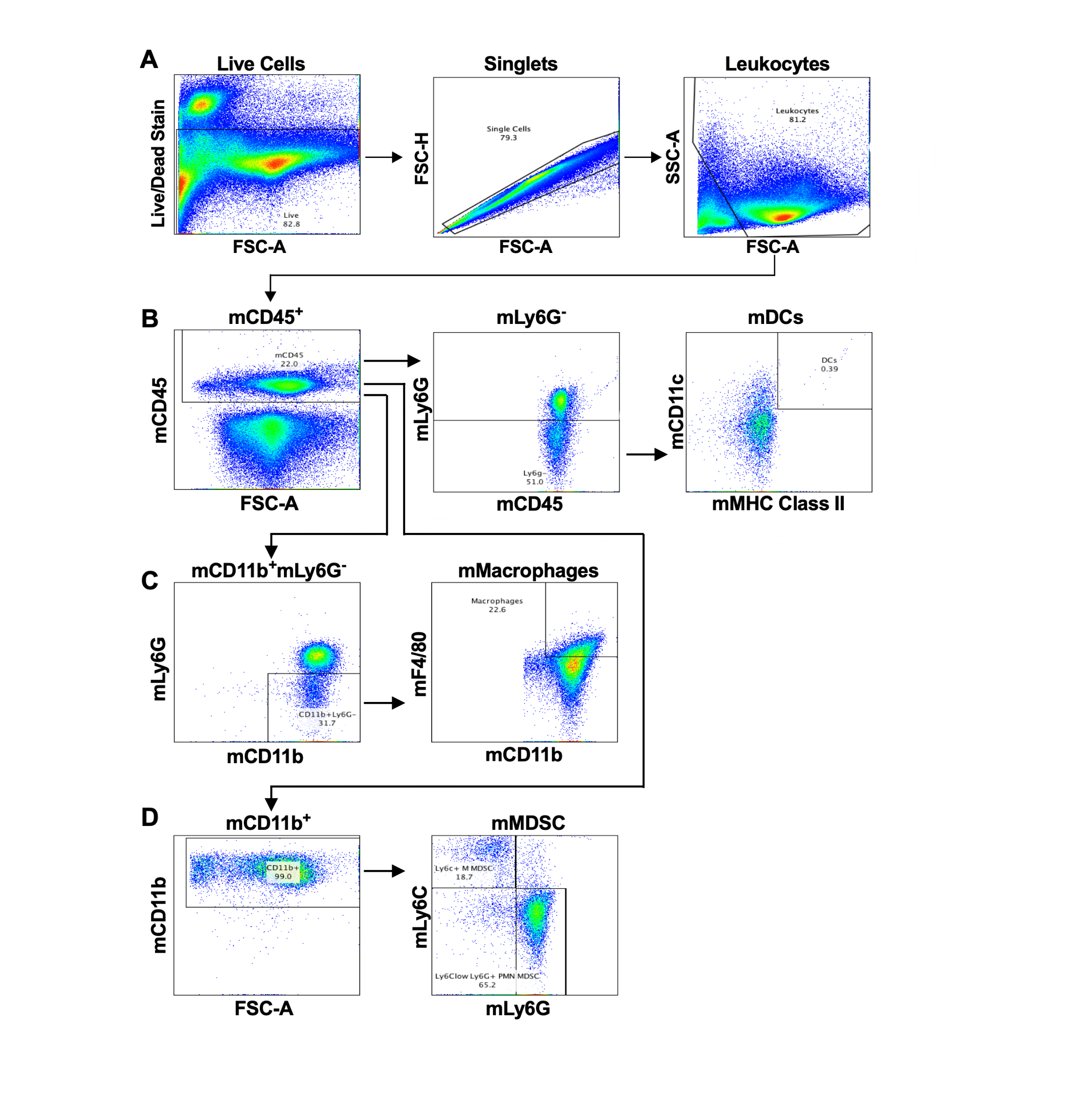


**Supplementary Figure 3: Gating strategy used to identify mouse leukocyte populations*.* (a**-**d)** Mouse leukocyte subsets were immunolabelled with mAbs and analysed by flow cytometry. **(a)** Live cells were gated based on forward scatter-area (FSC-A) and Zombie NIR staining. Singlets were gated based on FSC-A and forward scatter-height (FSC-H). Mouse leukocytes were gated using FSC-A and side scatter-area (SSC-A). **(b)** mCD45^+^ leukocytes were then identified before gating mCD45^+^ and mLy6G^-^ cells to identify mMHC class II^+^ mCD11c^+^ DCs, **(c)** or mCD11b^+^ and mLy6G^-^ cells to identify mCD11b^+^mF4/80^+^ macrophages **(d)** or FSC-A and mCD11b^+^ cells to identify mLy6G^+^mLy6C^lo^ PMN-MDSCs and mLy6G^-^mLy6C^+^ M-MDSCs.


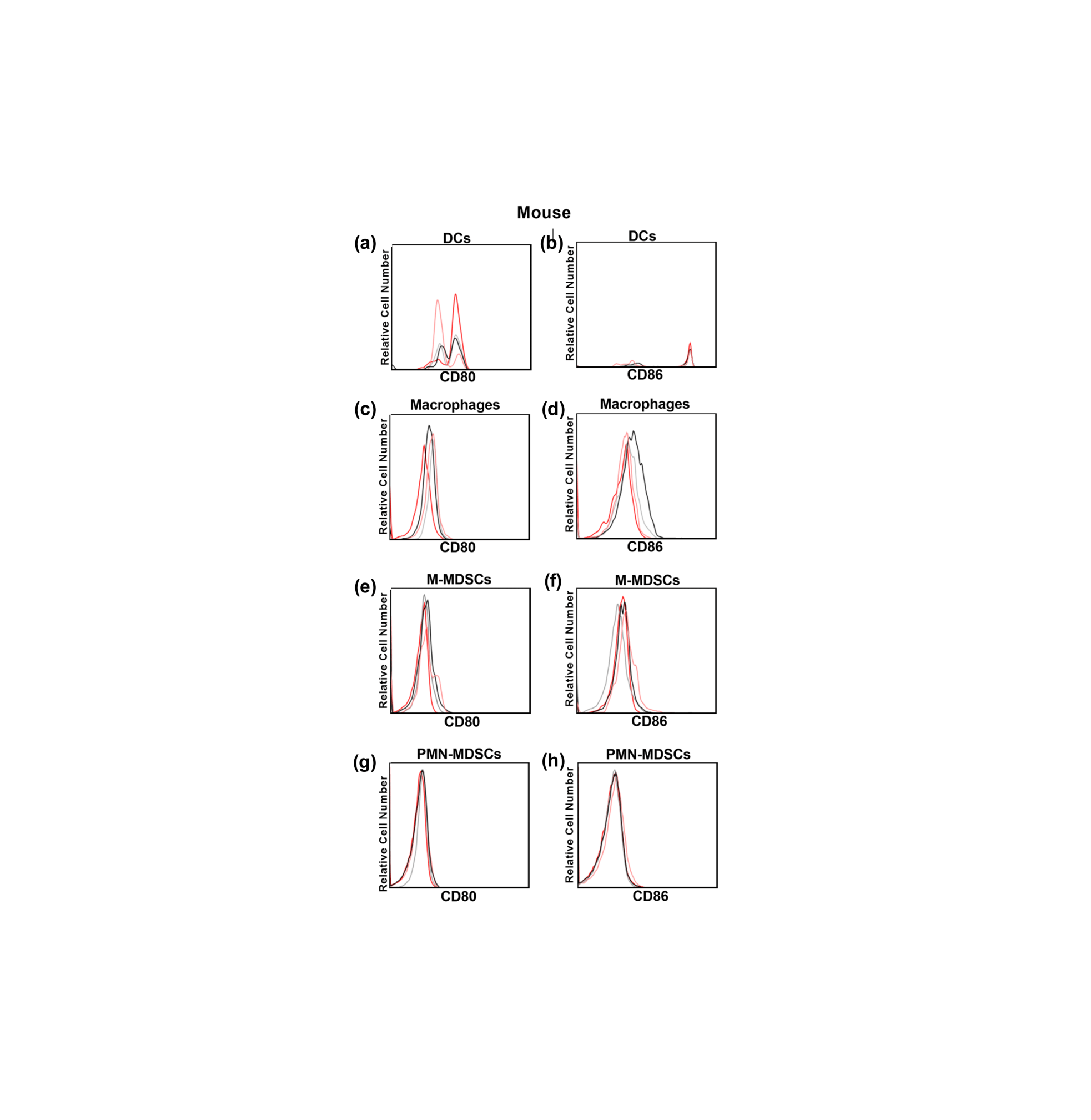


**Supplementary Figure 4: Cell surface expression of CD80 and CD86 on mouse leukocyte cells. (a-h)** Mouse leukocyte subsets from humanised mice treated with saline (black line), GFP (grey line), anti-mP2X7 Nb (red line) or anti-m/hP2X7 Nb (pink line) were immunolabelled with mAbs and relative amounts of cell surface CD80 and CD86 expression on each cell type examined by flow cytometry. Histograms displaying cell surface expression of **(a)** CD80 and **(b)** CD86 on mouse DCs, **(c)** CD80 and **(d)** CD86 on mouse macrophages, **(e)** CD80 and **(f)** CD86 on mouse M-MDSCs and **(g)** CD80 and **(h)** CD86 on mouse PMN-MDSCs (gated as per Supplementary figure 3). **(a-h)** Histograms representative of spleen cells from the same mouse for each treatment group.

**
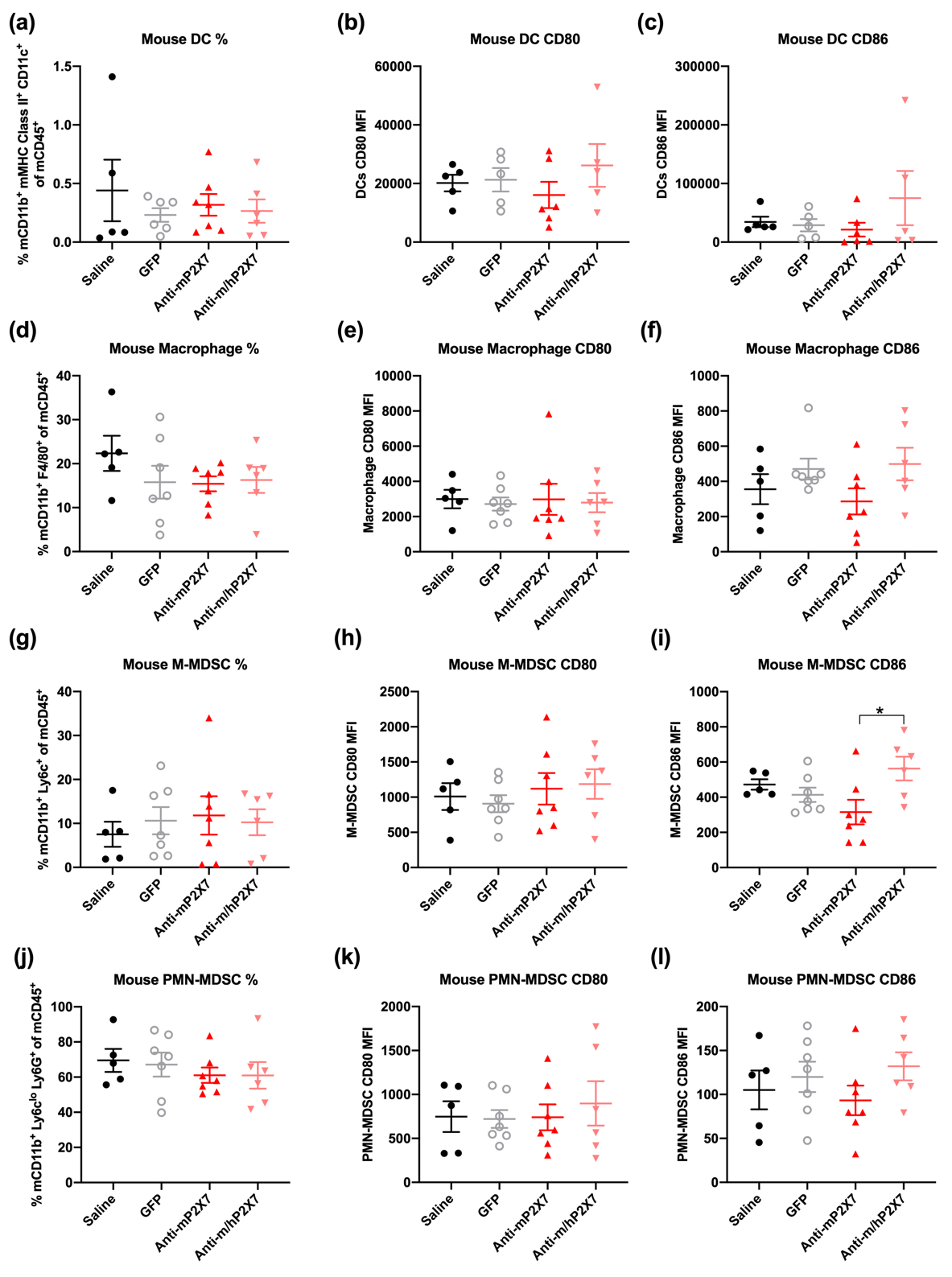
**

**Supplementary Figure 5: The anti-P2X7 Nbs did not alter mouse leukocyte proportions in the spleens of humanised mice at endpoint. (a-l)** Spleens from mice treated with saline (*n* = 5), GFP (*n* = 5-7), anti-mP2X7 Nb (*n* = 6-7) or anti-m/hP2X7 Nb (*n* = 6) were collected at humane (disease) or experiment (Day 70) endpoint and mouse leukocytes subsets and CD80 and CD86 expression were analysed by flow cytometry. **(a)** Proportions of mMHC class II^+^mCD11c^+^ DCs were identified before determining cell surface expression of **(b)** CD80 and **(c)** CD86 on DCs. **(d)** Proportions of mCD11b^+^mF4/80^+^ macrophages were identified before determining **(e)** CD80 and **(f)** CD86 on macrophages. **(g)** Proportions of mouse mLy6G^-^mLy6C^+^ M-MDSCs were identified before determining **(h)** CD80 and **(i)** CD86 on M-MDSCs. **(j)** Proportions of mLy6G^+^mLy6C^lo^ PMN-MDSC were identified before determining **(k)** CD80 and **(l)** CD86 on PMN-MDSCs. **(a-l)** Data are represented as mean ± SEM. Symbols represent individual mice. Data are from two independent experiments. Significance was assessed by the **(a, b, d, g-l)** one-way ANOVA with a Tukey’s post-test **(c ,e, f)** or Kruskal-Wallis test. * *p* < 0.05.

**
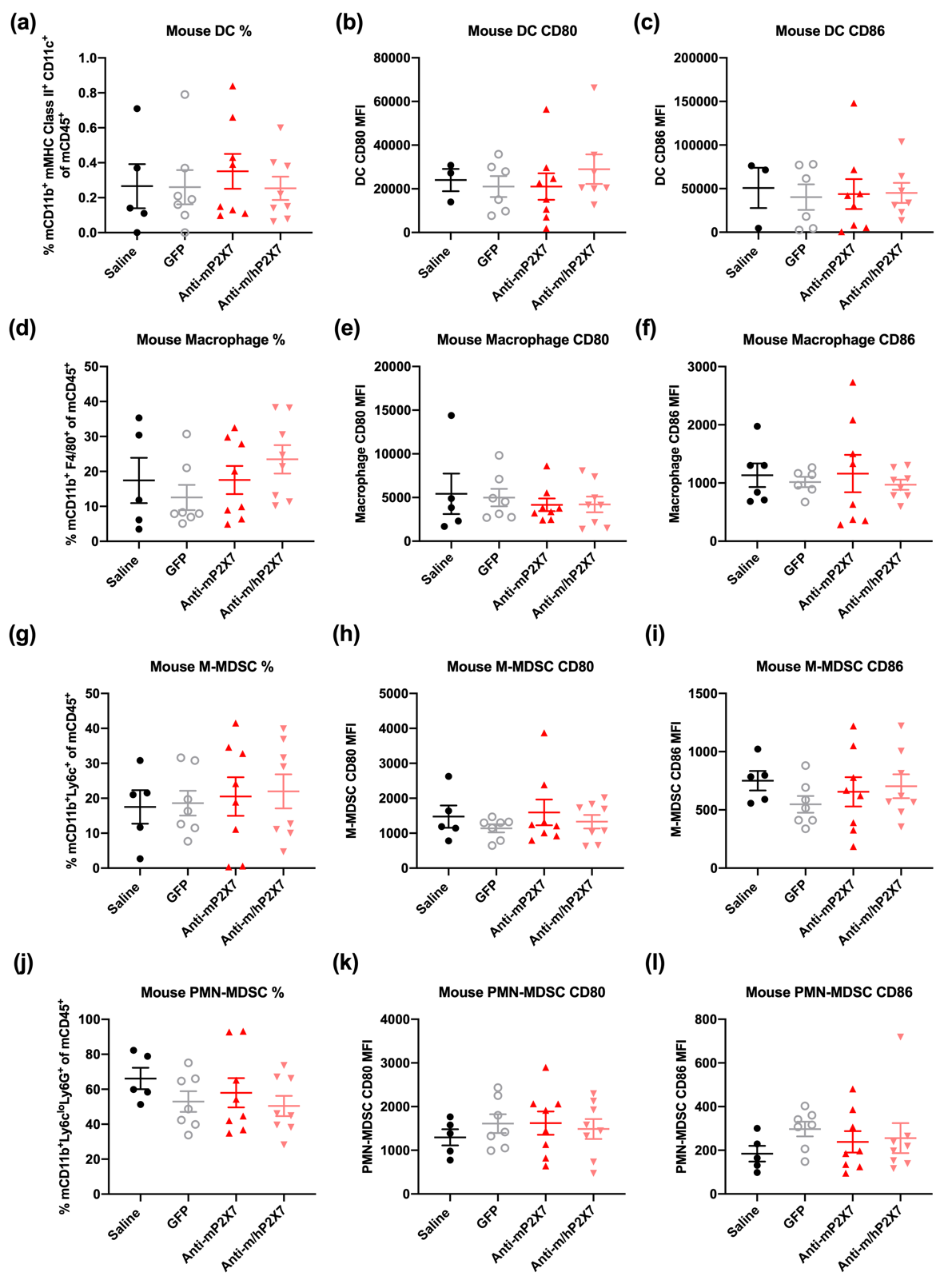
**

**Supplementary Figure 6: The anti-P2X7 Nbs did not alter mouse immune cell proportions in the livers of humanised mice at endpoint. (a-l)** Livers from mice treated with saline (*n* = 3-5), GFP (*n* = 6-7), anti-mP2X7 Nb (*n* = 8) or anti-m/hP2X7 Nb (*n* = 8) were collected at humane (disease) or experiment (Day 70) endpoint and mouse leukocytes subsets and CD80 and CD86 expression were analysed by flow cytometry. **(a)** Proportions of mMHC class II^+^mCD11c^+^ DCs were identified before determining cell surface expression of **(b)** CD80 and **(c)** CD86 on DCs. **(d)** Proportions of mCD11b^+^mF4/80^+^ macrophages were identified before determining **(e)** CD80 and **(f)** CD86 on macrophages. **(g)** Proportions of mLy6G^-^mLy6C^+^ M-MDSCs were identified before determining **(h)** CD80 and **(i)** CD86 on M-MDSCs. **(j)** Proportions of mLy6G^+^mLy6C^lo^ PMN-MDSC were identified before determining **(k)** CD80 and **(l)** CD86 on PMN-MDSCs. **(a-l)** Data are represented as mean ± SEM. Symbols represent individual mice. Data are from two independent experiments. Significance was assessed by the **(a, c, f, g, i-k)** one-way ANOVA **(b, d, e, h, l)** or Kruskal-Wallis test.

**
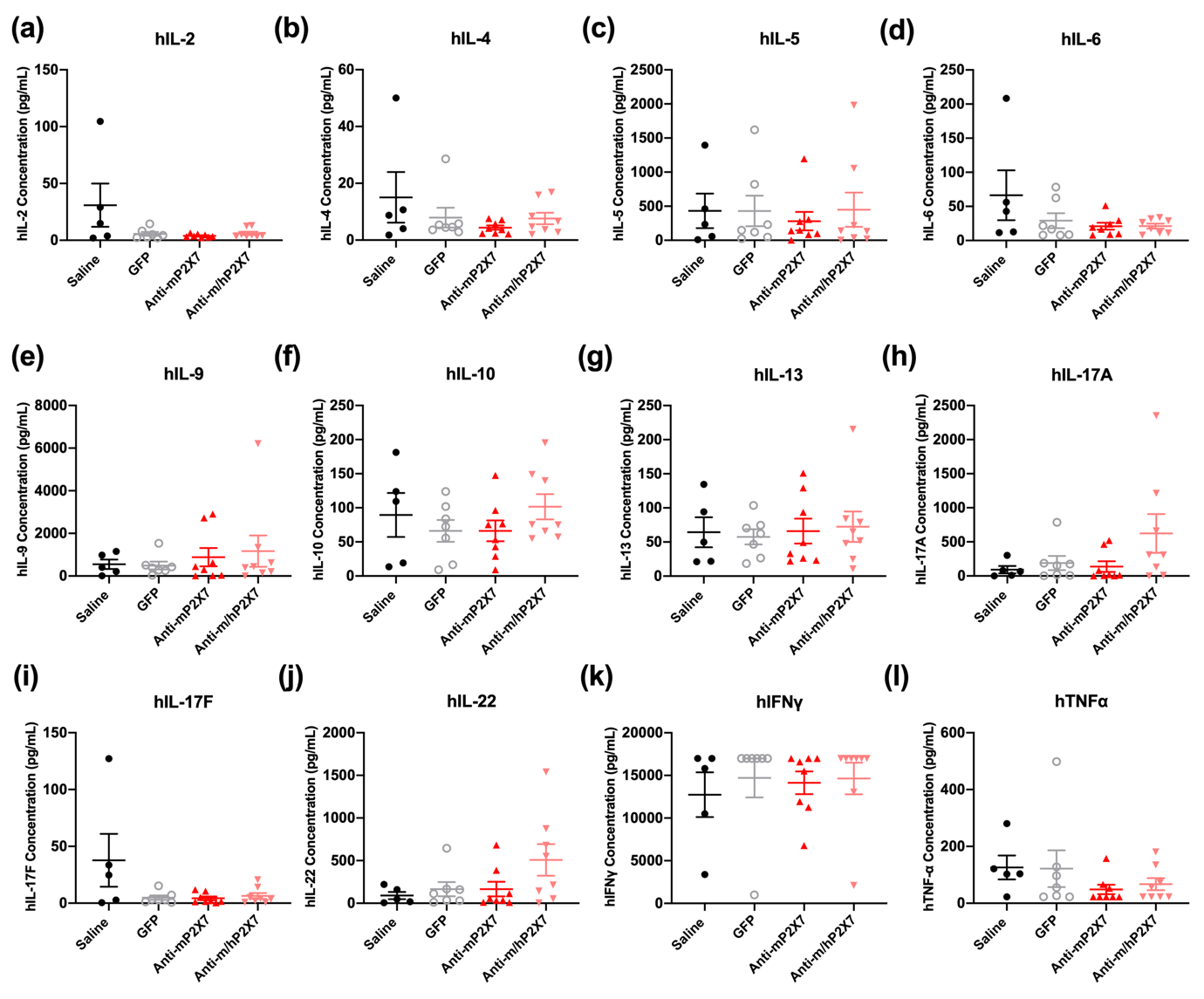
Supplementary Figure 7: The anti-P2X7 Nbs do not alter human cytokine concentrations in sera of humanised mice at endpoint. (a-i)** Sera from mice treated with saline (*n* = 5), GFP (*n* = 6-7), anti-mP2X7 (*n* = 8) or anti-m/hP2X7 (*n* = 8) were collected at humane (disease) or experiment (Day 70) endpoint. Concentrations of human (h) **(a)** IL-2, **(b)** IL-5, **(c)** IL-6, **(d)** IL-9, **(e)** IL-10, **(f)** IL-13, **(g)** IL-22, **(h)** TNFα and **(i)** IFNγ were analysed using a human T helper-1 LEGENDplex kit. (**a-i**) Data are represented as mean ± SEM. Symbols represent individual mice. Data is from one independent experiment. Significance was assessed by the **(a-e, g-l)** Kruskal-Wallis test or **(f)** one-way ANOVA with Dunn’s multiple comparison correction.

##### Supplementary Table 1: Anti-human mAbs used for the immunolabelling of cells for flow cytometry.

| Antibody | Clone | Fluorochrome^a^ | Dilution | Supplier^b^ |
| --- | --- | --- | --- | --- |
| CD3 | UHCT1 | BV711 | 1:50 | BD |
| CD4 | RPA-T4 | PerCP-Cy5.5 | 1:10 | BD |
| CD8 | RPA-T8 | PE-Cy7 | 1:50 | BD |
| CD19 | HIB19 | APC | 1:20 | BD |
| CD25 | M-A251 | PE | 1:20 | BD |
| CD39 | TU66 | APC | 1:20 | BD |
| CD45 | HI30 | FITC | 1:20 | BD |
| CD56 | MY31 | PE | 1:20 | BD |
| CD127 | HIL-7R-M21 | BV421 | 1:20 | BD |
| CD161 | HP-3G10 | BV605 | 1:50 | BD |
| TCR Vα24-Jα18 | 6B11 | PE-Cy7 | 1:50 | BioLegend |

^a^BV; Brilliant violet, PerCP-Cy; peridinin chlorophyll protein-cyanine, PE-Cy; phycoerythrin-cyanine, APC; allophycocyanin, PE; phycoerythrin, FITC; fluorescein isothiocyanate.

^b^BD Biosciences (San Diego, USA); BioLegend (San Diego, USA).

##### Supplementary Table 2: Anti-mouse mAbs used for the immunolabelling of cells for flow cytometry.

| Antibody | Clone | Fluorochrome^a^ | Dilution | Supplier^b^ |
| --- | --- | --- | --- | --- |
| CD11b | M1/70 | BV421 | 1:50 | BD |
| CD11c | HL3 | BV711 | 1:50 | BD |
| CD45 | 30-F11 | PerCP | 1:20 | BD |
| CD80 | 16-10A1 | BV750 | 1:20 | BD |
| CD86 | GL1 | PE-Cy7 | 1:20 | BD |
| F4/80 | T45-2342 | BV605 | 1:50 | BD |
| Ly-6C | 1G7.G10 | PE | 1:20 | Miltenyi |
| Ly-6G | 1A8 | FITC | 1:20 | Miltenyi |
| MHC Class II | M5/114 | APC | 1:20 | Miltenyi |

^a^BV; Brilliant violet, PerCP; peridinin chlorophyll protein, PE-Cy; phycoerythrin-cyanine, PE; phycoerythrin, FITC; fluorescein isothiocyanate and APC; allophycocyanin.

^b^BD Biosciences; Miltenyi Biotec (Bergisch Gladbach, Germany).
